# Antioxidants Protect Keratinocytes against *M. ulcerans* Mycolactone Cytotoxicity

**DOI:** 10.1371/journal.pone.0013839

**Published:** 2010-11-04

**Authors:** Alvar Grönberg, Louise Zettergren, Kerstin Bergh, Mona Ståhle, Johan Heilborn, Kristian Ängeby, Pamela L. Small, Hannah Akuffo, Sven Britton

**Affiliations:** 1 Molecular Dermatology, Department of Medicine, Center for Molecular Medicine, Karolinska Institute, Stockholm, Sweden; 2 Department of Dermatology and Venereology, Karolinska University Hospital, Stockholm, Sweden; 3 Department of Clinical Microbiology, Karolinska University Hospital, Stockholm, Sweden; 4 Department of Microbiology, Tumor and Cell Biology, Karolinska Institute, Stockholm, Sweden; 5 Department of Microbiology, University of Tennessee, Knoxville, Tennessee, United States of America; 6 Unit of Infectious Medicine, Karolinska University Hospital, Stockholm, Sweden; Texas A&M University, United States of America

## Abstract

**Background:**

*Mycobacterium ulcerans* is the causative agent of necrotizing skin ulcerations in distinctive geographical areas. *M. ulcerans* produces a macrolide toxin, mycolactone, which has been identified as an important virulence factor in ulcer formation. Mycolactone is cytotoxic to fibroblasts and adipocytes in vitro and has modulating activity on immune cell functions. The effect of mycolactone on keratinocytes has not been reported previously and the mechanism of mycolactone toxicity is presently unknown. Many other macrolide substances have cytotoxic and immunosuppressive activities and mediate some of their effects via production of reactive oxygen species (ROS). We have studied the effect of mycolactone in vitro on human keratinocytes—key cells in wound healing—and tested the hypothesis that the cytotoxic effect of mycolactone is mediated by ROS.

**Methodology/Principal Findings:**

The effect of mycolactone on primary skin keratinocyte growth and cell numbers was investigated in serum free growth medium in the presence of different antioxidants. A concentration and time dependent reduction in keratinocyte cell numbers was observed after exposure to mycolactone. Several different antioxidants inhibited this effect partly. The ROS inhibiting substance deferoxamine, which acts via chelation of Fe^2+^, completely prevented mycolactone mediated cytotoxicity.

**Conclusions/Significance:**

This study demonstrates that mycolactone mediated cytotoxicity can be inhibited by deferoxamine, suggesting a role of iron and ROS in mycolactone induced cytotoxicity of keratinocytes. The data provide a basis for the understanding of Buruli ulcer pathology and the development of improved therapies for this disease.

## Introduction

*Mycobacterium ulcerans* is the causative agent of necrotizing skin ulcerations known as Buruli ulcer (BU) in many West and central African countries and in Australia [1]. *M. ulcerans* produces a macrolide toxin, mycolactone, which has been identified as an important virulence factor in ulcer formation. Mycolactone is a 12-membered macrocyclic polyketide in which a second highly unsaturated polyketide side-chain is attached via an ester linkage. The natural variants of mycolactone share the core ring structure but vary in their unsaturated polyketide side chain [2], the core structure being substantially less toxic than the intact molecule [3]. Infection with a strain of *M. ulcerans* expressing mycolactone is associated with cell death and extracellular infection and pathology in a guinea pig model while a mycolactone negative mutant produces an intracellular granulomatous inflammatory infection similar to that of other mycobacterial species [4]. Mycolactone is cytotoxic to fibroblasts and adipocytes in vitro and has modulating activity on immune cell functions. Fibroblasts undergo apoptotic cell death after 3–5 days when exposed to mycolactone in vitro [5]. Adipocyte cell death after *M. ulcerans* infection involve direct necrosis caused by mycolactone as well as indirect apoptosis [6].

In the present study we have explored mycolactone effects on human primary skin keratinocytes in vitro. These cells are instrumental in the wound repair process by forming a protective epithelial barrier over the wound bed. Our finding is a concentration and time dependent reduction in cell numbers after exposure of keratinocytes to mycolactone . The mechanism of mycolactone toxicity is presently unknown. However, many other macrolide substances have cytotoxic and immunosuppressive activities and mediate some of their effects via production of reactive oxygen species [7], [8], [9], [10], [11], [12], [13], [14], [15], [16]. We therefore tested the hypothesis that the mycolactone effect on keratinocytes is mediated by reactive oxygen species and thus would be amenable for prevention by antioxidant treatment. To this end we have tested different antioxidants for effects on mycolactone mediated reduction of keratinocyte cell numbers.

## Materials and Methods

### Reagents

Mycolactone A/B was isolated and purified as described [17] and provided by PL Small. A 5 mg/ml solution in ethanol was prepared and stored at −20°C, protected from light. The antioxidants catalase (C3155), deferoxamine (D-9539), and Tiron (Fluka 89460) were from Sigma Aldrich (Stockholm, Sweden). TEMPOL and Trolox were obtained from Biomol International, L.P. (Plymouth Meeting, PA). CM-H2-DCFDA was obtained from Invitrogen (Stockholm, Sweden) and H_2_O_2_ from Sigma Aldrich.

### Cell culture

Human primary adult skin keratinocytes were obtained from Karocell AB (Stockholm, Sweden) and cultured in keratinocyte growth medium (Karocell AB) at 5% CO_2_, 37°C. The cells were detached by treatment with 0.05% trypsin-EDTA (Gibco, Invitrogen, Stockholm, Sweden) followed by trypsin inhibitor (Cascade Biologics, Invitrogen). The cells were used after 3–5 passages.

### Cell growth assay

Cell growth experiments were performed in 96-well plates by seeding 2000–10000 cells in 100 µl growth medium. The next day, the medium was removed and replaced by 80 µl fresh medium containing antioxidants. After 30 min at 5% CO_2_, 37°C, mycolactone was added in 20 µl and the incubation was continued for 24–72 h. Viable cell numbers were quantitated by adding 10 µl of the live cell labelling agent WST-1 (Roche Diagnostics, Stockholm, Sweden) for 20 min at 37°C, or by replacing the medium with 100 µl 0.05% neutral red (N2889, Sigma Aldrich) in growth medium and incubating for 1 h 37°C. Neutral red was extracted in 100 µl 70% ethanol and the plates were read in a spectrophotometric plate reader (Molecular Devices, Sunnyvale, CA). The results are expressed as optical density (OD) at 450 nm for WST-1 and 550 nm for neutral red correcting for background absorbance at 650 nm.

### ROS production

Intracellular ROS production was measured by labelling of confluent keratinocyte monolayers with 5 µM of the oxidation sensitive fluorescent probe CM-H2DCFDA for 60 min. The medium was removed and replaced with growth medium containing mycolactone and fluorescence (485 nm excitation/535 nm emission) was measured in a fluorescence plate reader Victor (Wallac) after 30–60 min.

### Statistical analysis

All data reported are representative of 2–4 experiments with at least three replicates of each condition. Differences between treatments in individual experiments and for combined data from four independent experiments were tested for statistical significance using ANOVA and Student's t-test.

## Results

### Mycolactone reduces keratinocyte cell numbers

**Figure 1:**
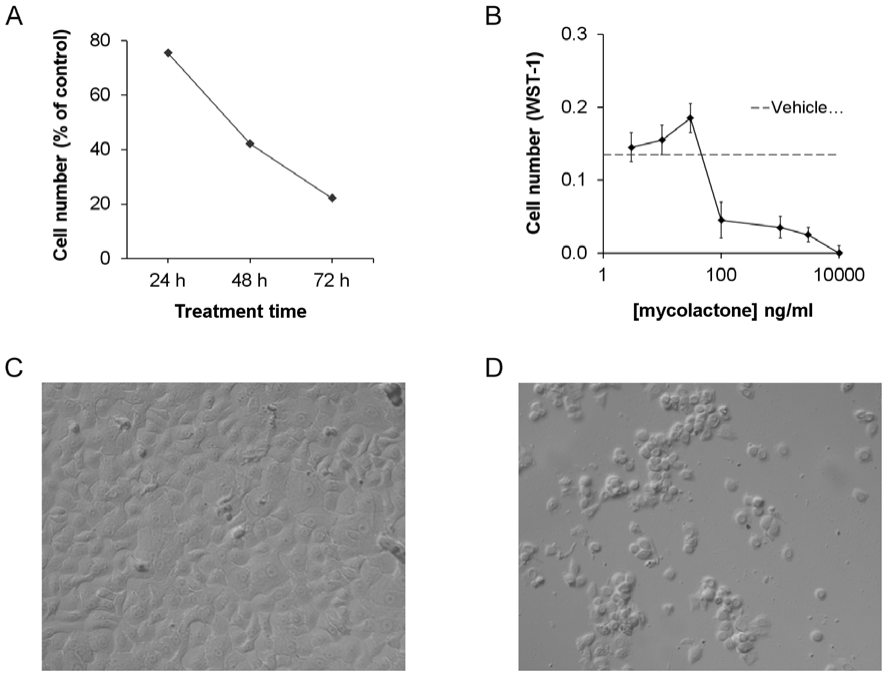
Mycolactone is cytotoxic for keratinocytes. Mycolactone was added to sub-confluent cultures of keratinocytes and (A) cell numbers were measured as optical density of WST-1after 24, 48 and 72 h of treatment with 1000 ng/ml of mycolactone, or (B) after 72 h at different concentrations of mycolactone. An asterisk indicates a significant (p<0.05) reduction in WST-1 labeling as compared to the corresponding medium control. Cell cultures of untreated controls (C) and cultures treated with 300 ng/m mycolactone (D) were photographed after 48 h. Data are from one representative experiment showing means and SD of triplicates.

Subconfluent keratinocytes were treated with mycolactone at a concentration between 1 and 10000 ng/ml for 24–72 h. After 24 h, cell rounding and detachment were observed in cultures containing >30 ng/ml mycolactone and a decrease in cell numbers was observed after 48–72 h (Figure 1A). At concentrations below those for which overt cytotoxicity occurred, a slight but statistically non-significant increase in cell numbers could was observed. When the concentration was increased to 100–300 ng/ml, there was reduction in cell numbers of approximately 70%. After 72 h of culture in 10,000 ng/ml of mycolactone there was a complete elimination of viable, WST-1 reactive cells in the cultures (Figures 1B and 1D). H_2_O_2_, which was used as a positive control, caused an almost complete reduction in cell numbers at a concentration of >10 µg/ml (data not shown).

### Effect of mycolactone on ROS production

**Figure 2:**
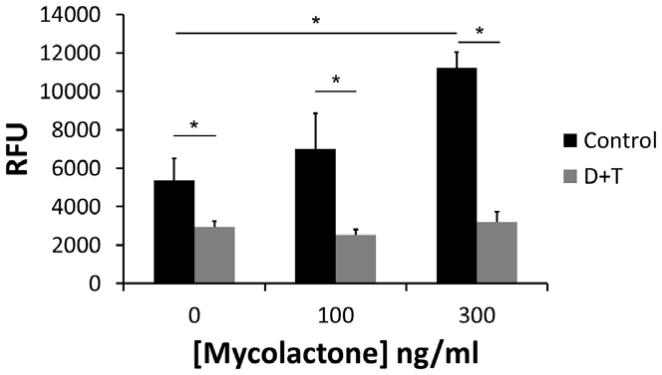
Mycolactone increases ROS production. Intracellular ROS production was detected by intracellular CM-H_2_DCFDA fluorescence in keratinocytes after 45 min incubation in control medium or with 100 and 300 ng/ml mycolactone. Parallel cultures were pretreated for 30 min with a combination of deferoxamine (100 µM) and TEMPOL (200 µM) (D+T) before CM-H_2_DCFDA and addition of mycolactone. Asterisks indicate significant (p<0.05) differences in fluorescence between treatments and their corresponding control in one representative experiment with four to six replicates (means and SD).

Effects of mycolactone on keratinocytes was similar to that of H_2_O_2_ in two respects; (i) there was a tendency (statistically non-significant) that low concentrations increased cell numbers, and (ii) the concentration response curve showed a steep reduction in cell numbers over a relatively narrow range, suggesting a threshold dependent cytotoxic effect. Using an intracellular probe, CM-H_2_DCFDA, which becomes fluorescent when reacting with free radicals, we could demonstrate increased fluorescence after 30–60 min of treatment of keratinocytes with a cytotoxic concentration (300 ng/ml) of mycolactone (Figure 2). The increase in fluorescence was prevented by addition of a combination of antioxidants; deferoxamine and TEMPOL. As positive control we tested H_2_O_2_, which significantly increased CM-H_2_ DCFDA fluorescence and this could be prevented by catalase (data not shown). These results suggest that mycolactone, similar to other macrolides, increases oxidative stress levels in epithelial cells and this could be responsible for its toxic effect on keratinocytes.

### Antioxidants prevent mycolactone toxicity

**Figure 3:**
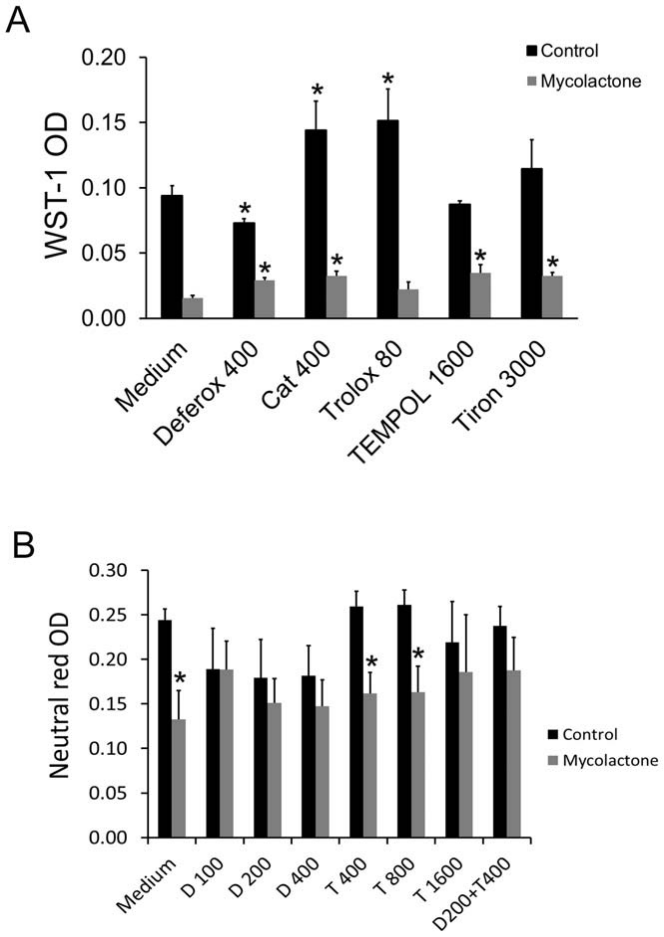
Antioxidant protection against mycolactone cytotoxicity. (A) Antioxidants were added 30 min before mycolactone (300 ng/ml) and the incubation was continued for 48 h when cell numbers were determined by measuring WST-1color at 450 nm. Data represent means of triplicate determinations and standard deviations from one representative experiment out of two performed (*, p<0.05, one way ANOVA and students t-test). (B) Deferoxamine (D) and TEMPOL were added at different concentrations alone or in combination 30 min before mycolactone (300 ng/ml) and the incubation was continued for 48 h when cell numbers were determined by measuring neutral red uptake Concentrations are in µM for all substances except catalase which is in U/ml. Data shown are means and SEM of four experiments (*, p<0.05, ANOVA and students t-test). TEMPOL 1600 µM was not included in the statistical analysis (N = 2).

We next investigated whether ROS was involved in mycolactone cytotoxicity by determining whether addition of catalase could prevent the effect of mycolactone on keratinocytes. In addition we used cell penetrating anti-oxidants such as (i) O_2_^−^ scavenging superoxide dismutase mimetics, TEMPOL and tiron, (ii) the iron chelating substance deferoxamine, which prevents production of OH^−^ via the Fenton reaction by, and (iii) a water soluble vitamin E analogue, Trolox. The antioxidants were used at concentrations reported to affect ROS production in other studies. Figure 3A shows that deferoxamine decreased, while catalase and trolox increase cell numbers in the control cultures. All anti-oxidants except trolox caused a partial but statistically significant protection against mycolactone cytotoxicity as indicated by the higher WST-1 staining in the antioxidant treated cultures. In order to exclude the possibility that antioxidant interference with the redox active substrate in the WST-1 reagent disturbed the analysis, cell quantitation with neutral red was performed, and the effects of deferoxamine and TEMPOL were investigated further. Deferoxamine at 100–400 µM abolished the cytotoxicity of mycolactone (Figure 3B) while TEMPOL had no effect at 400 µM and 800 µM. TEMPOL at 1600 µM showed significant protection in two out of three experiments. It was not possible to distinguish any additional effect on cytotoxicity if 400 µM TEMPOL was added to 200 µM deferoxamine.

## Discussion

We have demonstrated that the *M. ulcerans* produced toxin mycolactone is cytotoxic for human keratinocytes. Increased levels of intracellular ROS were found after treatment with mycolactone and this increase could be prevented by antioxidants. Pre-treatment with several different antioxidants showed partial protection against cytotoxicity. In particular, the iron chelating agent deferoxamine could provide complete protection against mycolactone mediated cytotoxicity.

Thus, keratinocytes, like fibroblasts and adipocytes [5], [6], [17], are sensitive to mycolactone toxicity in vitro. A recent study showed that keratinocyte stem cells and transit amplifying cells, differentiated by their adherence properties, undergo apoptotic cell death when exposed to 1–10 ng/ml mycolactone while the keratinocyte cell line, HaCaT, required a10-fold higher concentration [18]. These results clearly demonstrate that this bacterial toxin has effects on skin cells in vitro that may have a bearing on the observed tissue lytic effects in BU, since non-treated BU tissue contains biologically active mycolactone at approximately 10–100 µg/mg tissue [19].

The mechanisms whereby mycolactone mediates its toxic and inhibitory effects on different cells are incompletely known. We noted a similarity between the reactive oxygen species H_2_O_2_ and mycolactone in the way in which they affected keratinocyte cell numbers. We therefore tested the hypothesis that mycolactone increased ROS in keratinocytes. Using a fluorescent ROS sensitive probe we could demonstrate a concentration dependent increase in signal indicating increased production of ROS within 1 h of exposure to the toxin.

Drawing on the chemical structure of the core group of mycolactone we also speculated that mycolactone, like other macrolides, would cause cells to increase their production of ROS up to toxic levels. Cell toxicity dependent on ROS is described for the immunosuppressants cyclosporine A and FK506 [7], [10], [12], [14], [20], and antimycin A, an inhibitor of cell respiration at complex III [21], [22], [23]. Although it is unknown whether macrolides have a common mechanism responsible for increased production of reactive oxygen species, it is intriguing that a mycobacterial macrolide immunosuppressive toxin now also appears to share this property.

We consider it likely that mycolactone induced production of ROS would be cell dependent and may manifest itself differently and at different concentrations in different cell types, depending on their capacity to produce ROS and their internal antioxidant status. To this end, other epithelial cell lines like the embryonal kidney derived HEK293 line is reported as being resistant to mycolactone cytotoxicity [18]. Non-cytotoxic levels of ROS could be responsible for inhibitory effects on immune cells. For example, mycolactone inhibits IL-2 production by T cells [24], [25] and TNF-production by monocytes [26] . Such immunosuppressive effects by mycolactone would prevent an immune system mediated elimination of bacteria from infected tissue. Several studies of *M. ulcerans* infected patients have reported defective IFN-γ responses to *M. ulcerans* antigens [27], [28], [29], [30], [31], [32] as well as to other non-mycobacterial pathogens [33]. Down regulation of immune responses via local production of ROS has been identified as important mechanism for prevention of an autoimmune response in arthritis [34] and can be observed at the cellular level in macrophages as responsible for suppression of IL-2 production by T cells [35]. Inhibition of cytokine production by mycolactone in peripheral blood cells has been shown to occur at 100 ng/ml,a non-cytotoxic concentration for T cells, is manifested at the posttranscriptional level [36] and involves activation of the Src-family kinase Lck [37]. Interestingly, Lck activation by phosphorylation is dependent on redox regulation of cellular phosphatase activity and can be induced by treatment of T cells with H_2_O_2_ [38], [39].

A recent report describes that reactive oxygen species are involved in the defence against *M. ulcerans* infection based on reduced intracellular bacterial growth in keratinocytes *in vitro* in the presence of antioxidants [40]. Here, antioxidants prevented a reactive oxygen species dependent induction by *M. ulcerans* of the antimicrobial cathelicidin peptide fragment LL-37, which had an inhibitory effect on bacterial numbers in keratinocytes. However, a non-mycolactone producing strain of *M. ulcerans* was used in that study. The overall impact of reactive oxygen species production on *M. ulcerans* survival and growth in infected cells and tissue is not known. In general, intracellular forms of mycobacteria are considered relatively resistant to reactive oxygen species. *M. tuberculosis* elicits production of reactive oxygen species by host cells, and this contributes to their elimination. However, several detoxifying enzyme systems have evolved which help the bacteria to resist free radicals like H_2_O_2_, making some strains less susceptible to elimination via this mechanism [41], [42]. The *M. marinum* mel2 locus is important for its ability to resist reactive oxygen and nitrogen species in macrophages [43]. *M. ulcerans* has active catalase and superoxide dismutase [44] which may aid in resistance to host cell derived reactive oxygen species including those that may be generated via mycolactone.

The importance of the redox balance in wound healing is beginning to be recognized with the demonstration that low concentrations of reactive oxygen species stimulate wound healing and high concentrations are associated with chronic wounds [45]. There is no publicly available knowledge about the local and systemic oxidative status in BU patients. In two other mycobacterial diseases, leprosy and urogenital tuberculosis, there are signs of systemic oxidative stress that can be ameliorated by vitamin E supplementation [46], [47]. It is interesting to note that reactive oxygen species are able to suppress T cell functions [48] and regulate intracellular survival of mycobacteria [49]. Thus, at the same time as being an important part of the antibacterial defense system, reactive oxygen species may be tolerated by bacteria and contribute to disease. NO, a nitrogen based free radical, is implicated in the defense against mycobacteria [50] and has been investigated as a possible means to eliminate *M. ulcerans* and to improve BU healing. To this end, a small controlled trial of topical application of a acidified sodium nitrate as nitrogen oxide donor to BU showed significantly improved healing as compared with placebo [50]. Acidified sodium nitrate generates nitrogen oxide and effectively kills *M. ulcerans* in vitro [51]. Nitrogen oxide reacts with O_2_^−^ to form the reactive peroxynitrite radical which can contribute to killing of mycobacteria [52]. Preliminary data have shown that mycolactone cytotoxicity in vitro against primary keratinocytes is unaffected by addition of NO, inhibition of endogenous NO synthesis and by inhibition of the NADPH oxidase complex (unpublished results).

We found that, deferoxamine, which indirectly acts as an antioxidant, can mediate protection against mycolactone mediated cytotoxicity. Our demonstration that deferoxamine, which has iron chelating activity and thus prevents formation of OH^−^ via Fenton reaction, mediated complete protection, suggests that OH^−^ may be involved in the toxicity [53]. Alternatively, chelation of iron could have a direct effect on the cytotoxicity, implying that mycolactone cytotoxicity may be directly dependent on iron. Interestingly, excess iron is reported to promote *M. tuberculosis* infection in vitro [54] and is associated with increased susceptibility to tuberculosis [55], [56]. The role of iron in BU has to our knowledge not been investigated. Anaemia is common in most sub-Saharan African countries with BU, such as Benin [57]. However, a mutation in the gene for the iron export protein ferroportin, which is associated with mild anemia and a tendency to iron loading, is a common polymorphism in the African populations [58], [59]. Interestingly, ferroportin 1 is regulated by *M. tuberculosis* infection and is present in bacteria-containing phagosomes [60]. Further studies will be necessary to establish the iron status in patients with BU. [59]. Polymorphism in the gene for another iron transporter protein, Nramp1, is associated with susceptibility to BU [61]. The functional role of the allelic variant, which resides in exon 15 resulting in an Glu to Asn substitution, associated with increased susceptibility to BU, is unknown. A mechanistic interdependence between ROS, iron ion oxidation, and iron ion transport in the regulation of mycobacterial growth has been proposed by van Zandt et al., [60]. In the suggested model, Fe^2+^ is transported by Nramp1 into the phagosome, where it is oxidized to Fe^3+^, with production of OH^−^ as a consequence. The bacteria in the phagosome can then utilize Fe^3+^ for their intracellular growth. The role of ferroportin 1 is to export Fe^3+^ from the phagosome thus limiting the access of iron for bacteria residing in this compartment.

In conclusion, we have identified a novel mechanism whereby the *M. ulcerans* toxin mycolactone exerts its cytotoxic activity on keratinocytes. The mechanism is suggested to involve ROS and can be inhibited by the iron chelating agent deferoxamine. Whether these findings can be extended to encompass all effects of mycolactone operating during infection with *M. ulcerans* should be the subject of further investigations. Identification of safe and effective anti-toxins may provide a basis for development of more effective treatment of Buruli ulcers.
